# Qualitative Evaluation of a Complex Intervention to Improve Rheumatic Heart Disease Secondary Prophylaxis

**DOI:** 10.1161/JAHA.118.009376

**Published:** 2018-07-17

**Authors:** Clancy Read, Alison G. Mitchell, Jessica L. de Dassel, Clair Scrine, David Hendrickx, Ross S. Bailie, Vanessa Johnston, Graeme P. Maguire, Rosalie Schultz, Jonathan R. Carapetis, Anna P. Ralph

**Affiliations:** ^1^ Telethon Kids Institute University of Western Australia Western Australia Australia; ^2^ Charles Darwin University Darwin Northern Territory Australia; ^3^ University of Sydney University Centre for Rural Health Lismore New South Wales Australia; ^4^ Menzies School of Health Research Darwin Northern Territory Australia; ^5^ Medical School Australian National University Canberra Australian Capital Territory Australia; ^6^ Baker Heart and Diabetes Institute Melbourne Victoria Australia; ^7^ Centre For Remote Health Alice Springs Northern Territory Australia; ^8^ Perth Children's Hospital Perth Western Australia Australia; ^9^ Royal Darwin Hospital Darwin Northern Territory Australia

**Keywords:** acute rheumatic fever, adherence, chronic disease, quality improvement, rheumatic heart disease, systems of care, Rheumatic Heart Disease, Quality and Outcomes, Secondary Prevention

## Abstract

**Background:**

Rheumatic heart disease is a high‐burden condition in Australian Aboriginal communities. We evaluated a stepped‐wedge, community, randomized trial at 10 Aboriginal communities from 2013 to 2015. A multifaceted intervention was implemented using quality improvement and chronic care model approaches to improve delivery of penicillin prophylaxis for rheumatic heart disease. The trial did not improve penicillin adherence. This mixed‐methods evaluation, designed a priori*,* aimed to determine the association between methodological approaches and outcomes.

**Methods and Results:**

An evaluation framework was developed to measure the success of project implementation and of the underlying program theory. The program theory posited that penicillin delivery would be improved through activities implemented at clinics that addressed elements of the chronic care model. Qualitative data were derived from interviews with health‐center staff, informants, and clients; participant observation; and project officer reports. Quantitative data comprised numbers and types of “action items,” which were developed by participating clinic staff with project officers to improve delivery of penicillin injections. Interview data from 121 health‐center staff, 22 informants, and 72 clients revealed barriers to achieving the trial's aims, including project‐level factors (short trial duration), implementation factors (types of activities implemented), and contextual factors (high staff turnover and the complex sociocultural environment). Insufficient actions were implemented addressing “self‐management support” and “community linkage” streams of the chronic care model. Increased momentum was evident in later stages of the study.

**Conclusions:**

The program theory underpinning the study was sound. The limited impact made by the study on adherence was attributable to complex implementation challenges.

**Clinical Perspective**

**What Is New?**

- Creative and comprehensive approaches tailored for young people often living in disadvantaged and culturally diverse settings are needed to support their adherence to long‐term penicillin to prevent rheumatic heart disease; the chronic care model provides a helpful scaffold.
- This evaluation showed that health‐system–strengthening activities at primary care centers can fail to change outcomes for clients if strategies are insufficient to successfully engage the community and provide chronic disease self‐management support.
- Qualitative evaluation embedded in a randomized trial provides in‐depth understanding of outcomes and study context, including whether findings could be attributed to project design factors and how implementation occurred.

**What Are the Clinical Implications?**

- Use of incentives and removal of disincentives to receive penicillin injections were implemented, but their impact on measurable adherence was low because clients often lacked understanding of the value of the injections; this was attributable to impaired communication between healthcare providers and clients, in turn due to fundamental differences in culture and language, compounded by high staff turnover.
- Organizations that are culturally responsive and that support continuous learning and quality improvement can achieve better outcomes for clients.
- Continuous quality‐improvement cycles to motivate change can take time (>12 months) before improvements are seen.
- Community‐led, codesigned models of care are now being piloted, and innovative adherence support tools such as smartphone applications are gaining traction.

## Introduction

Rheumatic heart disease (RHD) comprises damage to cardiac valves during episodes of acute rheumatic fever (ARF) following group A streptococcal infection. More than 33 million people have RHD globally, causing >10 million disability‐adjusted life‐years lost and >300 000 deaths annually.^1^ In June 2017, the World Health Organization executive board recommended that a resolution on ARF and RHD be put to the World Health Assembly in 2018 to call on nations to implement evidence‐based approaches to RHD control. To date, little research has been done on health systems approaches to improving RHD care and prevention.

In the Northern Territory (NT) of Australia, efforts to control RHD need to take into consideration important sociocultural and economic factors underpinning high disease rates. The major current focus of RHD control in Australian NT Aboriginal communities is secondary prevention, consisting of long‐term injections of long‐acting intramuscular benzathine penicillin administered every 4 weeks. This treatment is delivered by healthcare providers in primary care clinics serving local communities, supported by a centralized register‐based RHD control program. Delivery of secondary prophylaxis is challenging, with the majority of ARF and RHD clients receiving less than half their scheduled injections and less than a third receiving the recommended ≥80% (M. Fittock, Registered Nurse, NT RHD Control Program, unpublished data, 2011).^2^

To improve delivery of ARF/RHD secondary prophylaxis, a comprehensive health‐system–strengthening strategy was developed and evaluated in a stepped‐wedge, community, randomized trial in 10 sites in Australia's NT between 2013 and 2016 (RHDSP [Rheumatic Heart Disease Secondary Prophylaxis] trial).^3^, ^4^ The trial was powered to detect doubling of the proportion of clients getting ≥80% of scheduled penicillin injections from 20% to 40%. All sites received a multifaceted intervention using continuous quality improvement (CQI) processes and activities aligned with streams of the chronic care model (CCM). The CCM is an approach to chronic illness management in primary care settings to create an “informed, activated patient and a prepared, proactive practice team” by addressing health systems, delivery system design, decision support, clinical information systems, self‐management support, and community linkages.^5^ ARF and RHD fulfill the definition of chronic conditions because management includes a minimum of 10 years of antibiotic administration as secondary prophylaxis for ARF recurrences, as well as other long‐term regular medical interventions.^6^ The CCM appears to be highly suited to delivery of care for ARF and RHD.^7^

Key findings from the RHDSP trial, reported in detail elsewhere,^4^ indicated that there was high variation in delivery of penicillin prophylaxis for ARF and/or RHD between sites, and the intervention did not improve penicillin adherence; in fact, adherence was slightly lower during the intensive phase of the trial (126/304 [41%] receiving the target of ≥80% of scheduled injections) compared with baseline (141/304 [46%], not a statistically significant difference). Adherence at baseline was already better than expected at 46%, compared with ≈23% previously (M. Fittock, Registered Nurse, NT RHD Control Program, unpublished data, 2011). Although the trial did not make further overall improvements, the subset of patients in the most adherent category (≥90% of scheduled injections) significantly increased during the maintenance phase. With only 10 participating sites and multiple differing characteristics of clinics, including patient numbers per site, level of staff turnover, socioeconomic indicators, numbers of action items completed, number of intervention visits achieved by the project officers, and clinic governance structure, no statistically significant associations could be identified between clinic characteristics and adherence outcomes. The 6 community‐controlled organizations performed similarly to the 4 government‐run services.

Recognizing the methodological, pragmatic, and theoretical limitations of randomized trials in evaluating complex health interventions,^8^ a comprehensive mixed‐method evaluation was designed at the outset to ensure that the trial's findings would be understood in depth. The aim of this article is to report qualitative findings of the evaluation framework that were collected during the trial to understand factors that affected the trial's implementation and outcomes. These findings have implications broadly across diverse settings aiming to improve health systems approaches to RHD control.

## Methods

### Trial Design

NT Aboriginal community clinics were invited to participate; 10 sites (sites A–J) consented, providing 304 clients.^4^ The required sample size was 300 clients.^3^ Pairs of sites entered the trial in random order according to a stepped‐wedge design at 3‐month intervals. A stepped‐wedge trial is a type of cluster randomized trial in which clusters receive the intervention at different time points, in random order, and comparisons are made between trial phases rather than between intervention and control sites.^3^, ^9^ This approach was chosen because it allows all sites to receive the intervention by the end of the study. Trial phases comprised (1) baseline data collection (12 months), (2) a transition phase with commencement of intensive phase activities but exclusion of data from outcome analyses (3 months), (3) an intensive phase (12 months), and (4) a maintenance phase (3–15 months depending on each site's start date).^3^ The variable duration of the maintenance phase is a consequence of the staggered commencement dates owing to the stepped‐wedge design.

The multifaceted intervention utilized quality‐improvement cycles and activities in all domains of the CCM including staff education and training. CQI processes aim to engage staff in understanding and responding to their own data and can be a powerful tool to motivate change in primary care settings.^10^ We previously showed incremental improvements in clinical care targets achieved in RHD management using CQI.^2^ Processes included proactive use of adherence data from each participating clinic to motivate healthcare providers and managers to work toward improved penicillin delivery targets, specifically, for more clients to achieve ≥80% of scheduled injections. Project officers presented clinics with quarterly data in simple graphics at face‐to‐face workshops throughout the intensive phase. To reach improved targets, each clinic developed and implemented a set of activities or *action items*.^3^, ^4^ Action items were compiled into *action plans*, aligned with the streams of the CCM, that would help the clinic to deliver the penicillin injections more effectively. Staff support included encouragement to undertake online training modules on ARF and RHD^11^ and on self‐management support and to integrate evidence‐based guidelines into daily clinical practice using online resources^6^ or smartphone applications.^12^ A quarterly study newsletter was produced to share ideas for strengthening the delivery of RHD prevention and to foster engagement with and ownership of the project by participating sites. It was circulated to participating sites and stakeholders throughout the study.

A program theory was developed, as we have described previously,^3^ to illustrate how the CCM would be translated into activities that would affect delivery of penicillin secondary prophylaxis for ARF and/or RHD clients by participating clinics. A *program theory* is a plausible model of how a program should work and provides a foundation on which a theory‐driven evaluation framework can be constructed.^13^ It described the *prescriptive assumptions* of the project (ie, What action is required to improve adherence to ARF/RHD secondary prophylaxis?) and *descriptive assumptions* (ie, Why will adherence be affected by these actions?). An example of an activity addressing the decision support stream of the CCM would be one that ensures all healthcare staff receive regular training in delivery of prophylaxis for RHD. An activity addressing the clinical information systems stream might be one that improves electronic systems to provide automated recalls for clients when prophylaxis is due. Both activities would be expected to lead to improved delivery of the required treatment.

To capture client perspectives, a nested focused ethnographic study (a description of cultural behavior focusing on ARF and RHD) was conducted at communities A, C, D, and G. These sites were chosen based on accessibility and language—different languages are spoken in different communities in the NT, and the researcher spoke 1 Aboriginal language. It used an exploratory approach to the phenomenon of the long‐term penicillin regimen to investigate client perspectives including what they understood and experienced of ARF and/or RHD, what their healthcare experiences were, how their age and culture influenced their self‐care, and what community‐ and clinic‐level factors facilitated their self‐care.

Approval was provided by the human research ethics committee of the NT Department of Health and Menzies School of Health Research (no. 2012‐1756) and the Central Australian Human Research Ethics Committee (no. 2013‐126). The data will not be made available to other researchers because interview participants consented for their interview material to be available to only nominated study investigators.

### Participants

Staff participants were eligible for interview if they were healthcare providers or ancillary staff working at participating clinics (doctors, nurses, Aboriginal health practitioners, clinic drivers [Aboriginal community members], receptionists), key stakeholders (NT RHD control program, working in remote health governance), or community informants (eg, teacher at a participating site). Two project officers employed by the project were also invited to be interviewed at the trial's conclusion. Individual written informed consent was sought to undertake and audio‐record interviews. Client participants were eligible to be interviewed if they resided at 1 of the 4 participating communities at which the ethnographic study was undertaken, were aged 5 to 35 years, and were currently prescribed intramuscular penicillin for ARF and/or RHD prophylaxis. An audio recording that explained the study and sought consent in 1 Aboriginal language was also used. Interviews were audio‐recorded and transcribed or were hand written if consent for audio recording was not provided.

### Data Sources

Data comprised semistructured interviews, project officer reports, observations, clinic action plan documents, and team meeting notes. In semistructured interviews, staff perspectives were sought at baseline regarding barriers to and enablers of delivering ARF/RHD prophylaxis and during the maintenance phase about their experience of the trial. The same staff members were interviewed serially if possible, but staff turnover meant this was not usually possible. In the nested ethnographic study, semistructured interviews and participant observations were carried out with young Aboriginal clients and their families at communities A, C, D, and G throughout the duration of the study.

The project officers were research nurses who visited the participating sites to support implementation of the intervention. They produced monthly reports and quarterly summaries including observation and informal discussion. They also documented clinic action plans and progress against individual action items. Individual action items were classified as completed, deleted, or ongoing, and for each, barriers and enablers were documented. Team meeting notes were generated from quarterly trial investigator meetings.

### Qualitative Analyses

**Figure 1:**
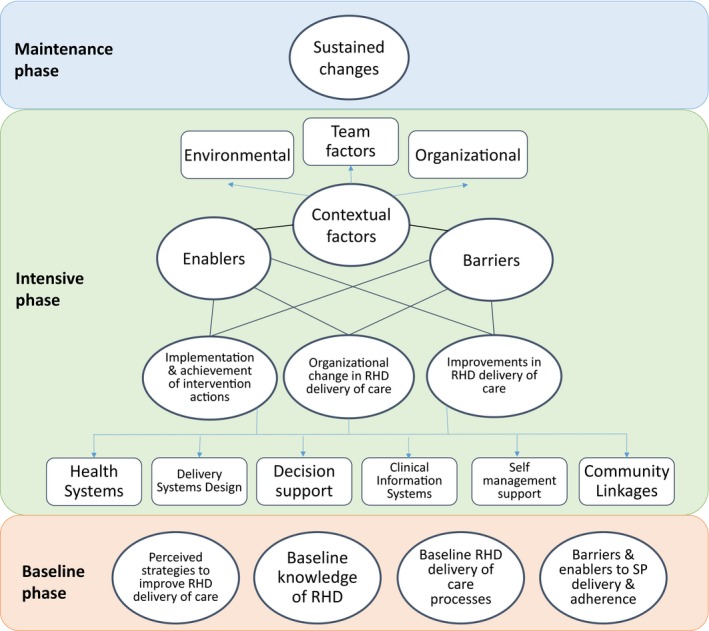
Coding map showing analysis codes applied to interview data from clinic staff members and key stakeholders. RHD indicates rheumatic heart disease; SP, secondary prophylaxis.

Analysis of interview data from clinic staff members and key stakeholders was undertaken by 3 analysts (2 postdoctoral qualitative researchers [C.R. and C.S.] and 1 mixed‐methods doctoral researcher [D.H.]) independent of the data‐collection processes and trial implementation, using a confirmatory approach. In other words, the analysis was guided by a specific hypothesis, and analysis codes were prespecified to answer defined research questions and then applied to the data, as appropriate (Figure 1).^14^ The hypothesis to be explored was whether the systems‐based intervention would improve adherence to secondary prophylaxis for RHD. For client data, responses were coded inductively and analyzed thematically by the qualitative doctoral researcher (A.G.M.) who conducted the interviews. For this component, an exploratory inductive approach was used because there was no preexisting hypothesis of how clients would respond to the intervention—that is, codes were developed de novo from the data without prespecified questions or assumptions. QSR International's NVivo 10 software was used to organize qualitative data.

### Evaluation

**Figure 2:**
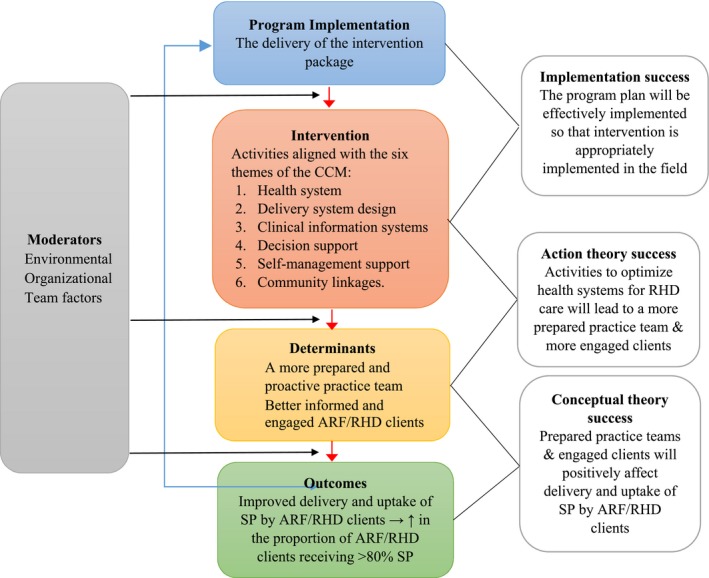
Evaluation framework. The central panel illustrates the interaction between implementation (delivery of the intervention to clients), intervention (the agents of change that affect determinants), determinants (change mechanisms), and outcomes (improved adherence). Underpinning theories (right panel) are action theory (the intervention's power to affect determinants) and conceptual theory (the determinant's ability to affect outcomes). External moderators to be accounted for (the environment, organization factors, and team factors) are shown at left. ARF indicates acute rheumatic fever; CCM, chronic care model; RHD, rheumatic heart disease; SP, secondary prophylaxis.

We developed a prespecified evaluation framework using the program theory described earlier, and based on implementation research concepts described elsewhere,^15^, ^16^ to determine what factors at health‐system, patient, and project levels affected the intervention's implementation and outcomes. The evaluation framework (Figure 2) sought to answer how and why the intervention might affect clinic performance by assessing the degree of project success at the levels of implementation, action theory (whether activities undertaken resulted in a more prepared and proactive practice team and more engaged clients), and conceptual theory (whether a more prepared and proactive practice team and more engaged clients influenced adherence). External moderators accounted for included environment, organization factors, and team factors. Evaluation measures were categorized under the headings of *efficiency, effectiveness, impact, relevance*, and *sustainability* (Table 1). Implementation process was measured according to fidelity to the trial protocol (development and implementation of relevant action plans), “dose” of the intervention (number of face‐to‐face visits by project officers to participating sites), “reach” (the number and quality of action items and comprehensiveness across the CCM themes), and acceptability of the project.

**Table 1:** Framework Used for Qualitative Data Collection and Analysis for Evaluation of the RHDSP Trial

Evaluative Criteria	Research Question	Indicator	Data Source
Efficiency: Degree to which inputs have been converted into outputs	To what extent did health centers change their delivery of RHD care to align with the systems‐based intervention?	Level of change: Number and quality of action items adopted; Streams of the CCM addressed by the action items	Health center action plans; Project officer reports (observation); Project officer interviews; Client interview data from ethnographic study; Team meeting notes; Baseline and maintenance‐phase interviews; Comparison of baseline report and maintenance‐phase reports; Comparison of quantitative findings from the analysis of the main trial with qualitative verification measures
Effectiveness: The extent to which the intervention's objectives were achieved	To what degree did adopting the systems‐based intervention improve processes of RHD care and adherence to secondary prophylaxis? Which elements of the intervention were most effective in activating change?	Level of improvement to processes: Association between components of CCM with primary outcome (SP adherence); Evaluation of program theory components, its causal processes, and expected outputs of the CCM	
Impact and relevance: The long‐term effects produced by the intervention and the extent to which the objectives of the intervention are consistent with beneficiaries’ needs	Did the intervention to strengthen the primary care‐level health system improve overall adherence to SP for RHD and minimize “days at risk?”	Measure of outcomes; Proportion of clients receiving 80% or more of scheduled penicillin injections over a minimum 12‐mo period (see outcome indicators for secondary indicators); Relation of outcome measures to implementation and intervention activities (completeness)	
Sustainability: The continuation of benefits from the intervention after the intervention has been completed	Which of the activities and streams of the CCM were sustained during maintenance phase?	Level of sustained change: Number of activities of CCM sustained; Number of streams of the CCM from which the activities were adopted sustained	Project officer reports (observation); Project officer notes of interaction with clinic staff during the maintenance phase
Process, fidelity, and acceptability: Whether program activities were implemented as intended and degree to which the intervention was implemented as expected	What was the fidelity, dose, and reach of the study? What was the acceptability of implementation of the intervention package, and of individual items? What were the barriers and enablers of implementation and of organizational change?	Inventory of CCM activities: Level of completeness in achieving activities of the CCM and as a whole; Level of acceptability of the implementation of the intervention; Factors moderating the implementation process (delivery of intervention); Factors moderating the process of organizational change (uptake of intervention)	Project officer reports (observation); Project officer interviews; Team meeting notes; Baseline and post‐intensive interviews; Project officer activity log (number of visits, time in field)
Overall performance: How well did the project achieve its goals?	What were the factors associated with success in achieving organizational and client level improvements in SP for RHD?	Contextual factors moderating transfer of inputs and activities into outputs (nonactivity enablers of effective change)	Project officer reports (observation); Project officer interviews; Client interview data from ethnography study; Team meeting notes; Baseline and maintenance‐phase interviews

CCM indicates chronic care model; RHD, rheumatic heart disease; RHDSP, Rheumatic Heart Disease Secondary Prophylaxis; SP, secondary prophylaxis.

Methods used to ensure robustness of the qualitative analyses included independent analysis of interview and observational data by qualitative data analysts who did not participate directly in conducting the trial; triangulation by using diverse sources of data, a number of collaborative investigators, and different methodological approaches; and *peer debriefing*, that is, ongoing dialogue among the project officers, investigators, and qualitative data analysts to ensure that information had not been misinterpreted.

### Role of the Funding Source

The study was funded by the Australian National Health and Medical Research Council and Wesfarmers Center for Vaccines and Infectious Diseases at Telethon Kids Institute. The funders had no role in the study design, data collection, analysis, interpretation, or writing of this report.

## Results

**Figure 3:**
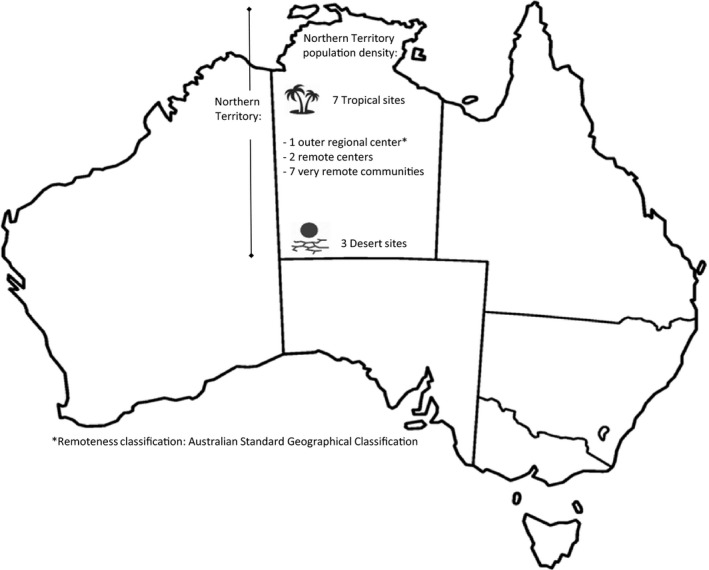
Map of Australia showing the Northern Territory and selected characteristics of participating communities.

Primary healthcare services participating in the RHDSP trial were located in 10 rural, remote, or very remote Aboriginal communities in the Australian NT (Figure 3). Consenting interview participants during the baseline phase comprised 107 clinic staff members working at the trial sites, 10 key informants, and 5 community stakeholders. During the maintenance phase, interviewees comprised 38 clinic staff members (24 of whom had also been interviewed during baseline) and 7 key informants (all of whom had also been interviewed during baseline). Within the nested ethnographic study, 72 clients (people with ARF and/or RHD or their family members) were interviewed. Fifty quarterly project officer reports provided field observation and data on monitoring of study progress. Ten documents, 1 from each site, reported on the development and implementation of action plans to improve the delivery of penicillin prophylaxis for clients with ARF and/or RHD.

### Efficiency

**Figure 4:**
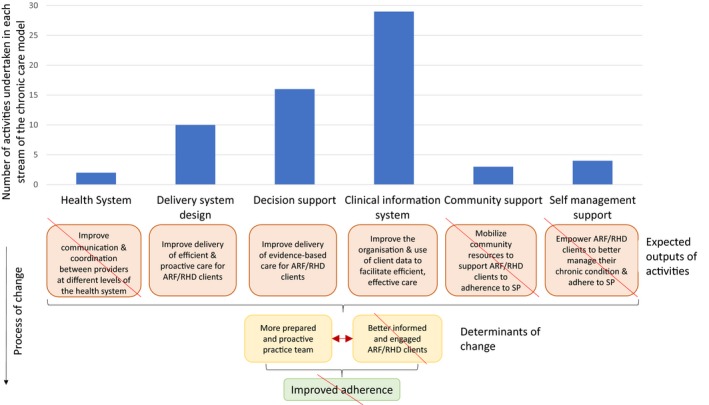
Distribution of activities across streams of the chronic care model affecting attainment of trial outcomes. ARF indicates acute rheumatic fever; Strike‐out, insufficient success; RHD, rheumatic heart disease.

During the transition and intensive phases of the trial (15 months), 252 action items were proposed for implementation by healthcare providers.^4^ Of these, 109 (43%) were completed (Tables 2 and 3, Figure 4). Of the completed items, 45 were categorized by project officers as *preparatory* and 64 as *core* actions. This categorization provided a way to help staff see momentum in progression of their action plan, working through small, manageable preparatory steps, as applicable, before being able to implement the core action. Completing core actions by the end of the intervention phase was challenging. As an example, one site devised an action item of hosting an RHD community event; preparatory actions included workshopping different ideas for the event, but the event had not occurred by the end of the intensive phase (although it did occur in the maintenance phase). There was variation among the 10 participating sites in the number of core action items completed (between 1 and 13) and in the ability of the accomplished actions to directly affect adherence; for instance, the action item “consult with the local school about the feasibility of injection administration at the school” did not lead to improved adherence because that school did not support delivery of injections, whereas a plan to “ensure that triage and other processes for clients are fast tracked” was likely to reduce the chance of a client leaving before receiving an injection. Some changes made by clinics were very visible, such as construction of a new cubicle in the waiting room to facilitate efficient injection delivery at one site.

**Table 2:** Action Items Completed During the Intensive and Maintenance Phases of the Study, According to CCM Streams

Participating Health Center	CCM Stream						Total
	Clinical Information Systems	Community Linkages	Decision Support	Delivery System Design	Health Systems	Self‐Management Support	
Intensive phase							
A	1	0	0	0	0	0	1
B	6	0	3	3	1	0	13
C	8	0	1	1	1	1	12
D	6	1	2	1	0	0	10
E	0	0	2	1	0	1	4
F	2	0	0	0	0	0	2
G	0	0	3	0	0	0	3
H	3	1	2	2	0	2	10
I	1	1	2	1	0	0	5
J	2	0	1	1	0	0	4
Total	29	3	16	10	2	4	64
Maintenance phase							
A	1	2	0	3	0	0	6
B	3	3	2	3	0	0	11
C	4	0	0	1	0	0	5
D	2	0	2	1	0	1	6
E	2	0	1	0	0	0	3
F	7	0	2	1	0	1	11
G	0	0	0	0	0	0	0
H	0	0	0	0	0	0	0
I	0	0	0	0	0	0	0
J	0	1	2	0	0	0	3
Total	19	6	9	9	0	2	45

CCM indicates chronic care model.

**Table 3:** Contextual Factors Affecting the Study's Causal Processes and Attainment of Outcomes

Categories Explored	Examples Identified Among Participating Health Centers^a^
Environmental	*Cultural disconnection between providers and consumers of health care (both language and conceptual differences)* *Insufficient mechanisms to support continuum of care for Aboriginal clients traveling between communities* *Complex and competing priorities within communities that distract attention from RHD and its importance (ie, underlying poverty and dysfunction)*
Organizational	*Competing priorities and high workload of health‐center staff* *High staff turnover in health centers* *Inadequate infrastructure to support quality improvement processes and system redesign* Occasional difficulties with communication within and between health centers and with the control program Culture of healthcare delivery that de‐emphasized positive health‐seeking behaviors among clients
Team factors	Poor knowledge and awareness of RHD and the need for penicillin prophylaxis among staff in some health centers Inconsistent senior leadership support for quality improvement

RHD indicates rheumatic heart disease.

a Most consistently identified factors shown in italics.

Almost half the completed core action items (29/64, 45%) were dedicated to the CCM theme of clinical information systems, followed by decision support (25%) and delivery system design (15%; Table 2). In contrast, the community linkages and self‐management support themes received little attention (6% each). According to the program theory we devised^3^ and previous studies using the CCM framework,^17^, ^18^ a breadth of activities across the CCM is required to adequately effect change; therefore, the intervention as implemented lacked reach and, consequently, was inefficient in affecting the determinants of “prepared and proactive practice teams” and “better informed and engaged clients” (Figure 4). This was likely to have been a major contributor to lack of achievement of that trial's primary outcome of a doubling in the proportion of clients getting ≥80% of scheduled penicillin injections.

A strength of the action items was that these were usually highly specific to context, having been devised by staff familiar with RHD program delivery in their setting who could recognize the gaps needing attention. However, many were fairly minor, unable to therefore collectively lead to genuine health system change.

### Effectiveness

Owing to the lack of reach of the intervention (ie, number and quality of action items and comprehensiveness across the CCM themes), effectiveness of the intervention was curtailed. Nevertheless, although the action items implemented did not lead to improved adherence overall, there were examples of changes in practice that had an observed positive effect on processes of care. Examples include development of client recall lists and updating of client records to streamline recall processes. More accurate patient data and improvements in the organization and use of patient data facilitated more efficient and effective care. Clinics were widely encouraged to start recalling patients for their next injection from day 21 after a previous injection to ensure that the next dose was achieved within the recommended 28‐day time frame. Informing clients at day 21 allows them up to a week to get to the clinic to be able to maintain adherence to a 28‐day regimen. Two clinics that changed the timing of recalls from 28 to 21 days found this to be an “easy mindset to change,” resulting in better understanding among staff that injections can be delivered safely before 28 days, recognition of the value of commencing the reminder process earlier to reduce “days at risk” of ARF recurrence, and increasing the delivery of opportunistic injections (ie, administering a penicillin dose if near the due date when the client attends the clinic for other purposes). Staff noted that the change meant they “had to ‘chase’ (clients) more often” but also that the change was seen as a “good reminder for staff that it [benzathine penicillin] is due.”

Increased staff training on ARF and RHD care, mostly through completion of RHD Australia online modules, addressed gaps in knowledge of ARF and RHD; however, time constraints were a major barrier to completing training modules. Regular presence of the project officers in clinics and the process of identifying where improvements to practice could be made had an observed positive impact on the attitudes of some clinic staff toward RHD. During the post–intensive phase feedback, for instance, acceptability and benefits of the trial were articulated by healthcare providers at some sites: “If not for this study, RHD would not be on the radar [in the clinic]—now it's on everyone's radar, both management and the other [partner] clinics” (healthcare provider).

Interviews with clients revealed that they had many questions about their condition and often felt powerless to ask them within the clinic environment. This was compounded by communication difficulties because of the need to operate in English (not the primary language of most clients, but interpreters are used infrequently) as well as in unfamiliar communication styles in health services. Yet there was a strong desire for, and a sense of exclusion from, true knowledge. A key theme to emerge from client interviews was that knowledge of ARF and RHD, and the reason for regular injections, was very limited. As an example, a young woman who had received penicillin for many years asked, “What were the injections for?” Another young woman with recently acquired ARF asked, “How can we stop that sickness?” The mother of a girl with RHD asked, “Where does it come from?” This questioning contrasted sharply with comments from clinic staff who thought they were providing effective education to clients, but were clearly failing: “How much education do you give people? I mean, I feel like we're just giving them a lot of information that they're paying lip service to understanding and listening to. Either they don't understand, or they don't want to listen, and that's their prerogative really. They expect us to create miracles and keep them alive, yet they're not doing their part of the bargain” (healthcare provider).

Aboriginal participants identified that focusing on language, place, and time could improve communication of health information. Use of their own languages was seen as being vital; if that were not possible—as is frequently the case when no local Aboriginal health practitioners, community health workers, or interpreters are available—then information should be delivered in way that would be understandable, drawing on and respecting traditional knowledge and using appropriate analogies rather than being based on a typical Western model of health communication. The place they wanted to receive information was their own space, not inside the clinic, which was often viewed as an alienating environment. Taking time—many discussions over months, with a consistent person—was also requested. When education about the need for adherence to penicillin was delivered by the ethnographic researcher (A.G.M.) according to these strategies, families gained a deep and urgent sense of responsibility to ensure their children received the treatment. On learning of the critical timing for the penicillin injection, a grandmother of a boy with ARF called the community clinic where her grandson was staying to ensure they would arrange for him to receive his next injection on time. Another grandmother, after learning of the recommended recall at 21 days to avoid late delivery of the penicillin injection, stated, “My daughter is looking after [boy with RHD]. I will ring her up and tell her about counting 21 days.”

These findings reinforce the conclusion that study implementation would have been more efficient if more activities had successfully addressed the community linkages and self‐management support themes of the CCM.

### Impact and Relevance

The study activities were often inconsistent with the perceived needs of providers. The most pressing needs of healthcare providers related to improved staffing levels, which the intervention was not designed to address. One healthcare provider commented, “All of these processes take thought and time and a lot of planning. And we just don't have it.” A healthcare provider at another site commented, “I was quite interested in the beginning and we could have done this, this, and this and could've got really involved in it, but as I said, just got so caught up in other things and it really just lost … yeah, it lost its drive, I guess.”

Regarding relevance of the intervention to people with ARF and/or RHD, it was difficult to discern the impact of the trial on clients. One client, in response to questioning about whether anything had changed about his ARF care, indicated that he had not observed any change at all other than the issue unrelated to the study itself of high staff turnover, stating, “There are new nurses.”

However, some patients did perceive improvement in access to injections outside the clinic via outreach services and reminders from clinics. A young woman responded, “They send me text messages and the nurse comes to see me at home, and if I am at school, she comes to the school and gives the injection.”

### Sustainability

Action plans continued to be implemented during the maintenance phase of the RHDSP trial, indicating potential for sustainability. The trial's primary end point, however, was evaluated during the intensive phase—which provided a more robust measure with 12 months of data across all sites—so processes that gained momentum later were not captured in that measure, and a longer time frame for implementing the study may have been beneficial. Forty‐five additional core action items were completed in the maintenance phase (Table 2). The CCM themes addressed by these later core action items were similar in scope to those in the intensive phase (clinical information systems, 19; delivery system design, 9; decision support, 9; self‐management support, 2; community linkages, 6). Site H, which achieved 2 core action items in the intensive phase, achieved 11 during the maintenance phase. Site A, which achieved only 1 core action item by the end of intensive phase, achieved another 6 during the maintenance phase. At the end of the trial, 71 action items remained ongoing (47 core, 24 preparatory).

At a high‐performing site that engaged well with the trial, a strong sense of ownership, pride, and sustainability relating to the project was evident. During a post–intensive phase feedback process, a nurse at this site stated of the project that “we got a sense of achievement because we were making the changes. Even though [the project officer] has gone, we don't need her to be ringing us up once per month because we are continuing to make changes ourselves.”

Data obtained from the focused ethnographic study highlighted the contrast between the stability of the local population, which stays on traditional lands with periodic travel between culturally linked locations, and the high churn of nonlocal healthcare providers who were often in the community as locum tenens staff for 2‐ to 6‐week periods. The constant change in clinic staffing reduced the potential for sustainability of this CQI intervention. In this environment, sustainability in project implementation could be provided by the community members themselves through better self‐management support and increased employment of Aboriginal people by health services. A project officer commented, “We had the light bulb moment a few months into the project where the only thing that doesn't change is the people in the community. So you're trying to encourage staff to do things better, but maybe what we need to work on is more on the community and patient side.”

### Process, Fidelity, and Acceptability

Regarding fidelity to the protocol, all participating sites successfully developed action plans and implemented at least 1 action item during the required period. The dose of the intervention delivered by the researchers varied between 7 of 15 monthly scheduled face‐to‐face visits at one site to 14 of 15 at another. One site declined to receive intervention visits for a period because of the clinic management's perception that there were excessive external visitors to the community

At some sites, there was limited understanding of the trial's objectives and the project officers’ role (which was to monitor and support rather than implement the action plans), and that affected study processes: “I suppose what we would like to see is maybe some more education from someone outside of this clinic for our rheumatic heart patients, and unfortunately you're just doing the research role” (healthcare provider).

In a project officer report, a discussion between a project officer and a clinic manager was documented, in which the clinic manager was quoted as saying, “We're just not happy with how the project is actually going and we don't understand why you're not doing it the way that we thought you were going to do it.”

Acceptability was influenced by staff attitudes toward the benefits of specific activities, particularly those relating to self‐management support and community linkages. Also, time required to implement RHD action plans was difficult to incorporate into daily workloads perceived as full given competing priorities such as acute care demands. Criticism of the trial included that it was too narrow, not adequately patient centered, and not sustainable because of staff shortages and high staff turnover at clinics: “Rheumatic heart disease isn't about secondary prophylaxis. It's about whole person and it's about prevention. So yeah, it's a very narrow research project” (healthcare provider).

Servicing the needs of external groups—such as other researchers, educators, visiting specialists, and government agencies—placed time pressures on and added workload to clinic staff, as highlighted by this healthcare provider's statement: “When we're at a clinic you have different people driving different programs. You've got rheumatic heart, then you've got the renal team come in. Then you've got sexual health, and they're all competing. They're all driving their own. They're passionate about their own programs. But on the coal face [front line], basically, it can get very competitive.”

A key enabler of implementation was the presence of staff members at clinics who displayed enthusiasm, willingness, engagement, and motivation for the trial. An association was apparent between the number of action items implemented and the presence of a well‐defined RHD coordinator role and/or a clinic manager who was supportive of ARF/RHD care and of the trial. At a clinic that implemented a high number of action items and had high adherence at baseline and throughout the trial, project officers noted the following characteristics: effective communication with the jurisdictional RHD control program and within the clinic; good engagement with the community, including knowledge and understanding of Aboriginal culture; and adequate allocation of time by clinic management for staff to complete action items, especially training and education.

### Overall Performance

Factors affecting trial outcomes are shown in Table 3, according to environmental, organizational, and team factors. The interplay among these factors creates high system complexity in the Australian NT context and provides further reasons for the intervention implementation challenges.

Factors found to impair clinics’ abilities to change practice to align with the intervention package were staffing issues, including high turnover, limited opportunities for handover, having to manage multiple portfolios, and unfilled positions; busy jobs with high work demands causing burnout, stress, low morale, and lack of time for staff education and other professional development activities; and chaotic clinic environments with internal communication failures and documented instances of tension and dispute. A nurse who coordinated the injections in her clinic alluded to the understaffing and unclear strategies for handover when she was going on leave and stated, “I hope someone takes the program on while I am away.” A project officer was asked to provide induction for a new staff member at one clinic and reported, “The agency nurse was not aware that one of her roles was RHD coordinator.” Although there were many occasions where individuals encouraged teams to make positive changes, efforts of individuals were impeded if the clinic environment was characterized by high staff turnover and high workload and a culture resistant to change.

In contrast, a key factor that facilitated organizational change and attainment of outcomes was effective clinic management. Positive changes implemented by one clinic manager to improve adherence for ARF and/or RHD clients included opening the clinic through lunch hours; providing opportunities for staff to shift portfolios, if they wished (eg, from acute to chronic care management); encouraging staff to identify areas they would like to improve with goal setting and provision of strategies to support goals; and providing positive feedback to staff.

## Discussion

We undertook a comprehensive evaluation of the RHDSP community randomized trial to improve the delivery of penicillin as secondary prophylaxis for Aboriginal people with ARF and/or RHD in Australia's NT. Findings reveal factors pertaining to the project design, implementation, and context that contributed to a lack of improved adherence. A chief project design factor identified was the relatively short time allocated in which to implement and measure the primary outcome. The chief implementation factor was that action items addressing the self‐management support and community linkage themes of the CCM proved very difficult to conceptualize, let alone achieve, and the number and quality of action items were insufficient to activate change. The chief contextual factors were staffing challenges in remote settings (understaffing and high turnover) and the sociocultural environment, characterized by cultural and language differences between healthcare providers and Aboriginal clients, resulting in impaired communication; ultimately, the health care being provided was not seen by the end users in this study (Aboriginal people) as meeting their needs.

The trial design underestimated the time needed to implement activities that could result in genuine change. The duration of the project implementation phase was 15 months. Although the stepped‐wedge design provided a 3‐month transition period before commencement of the 12‐month intensive phase of quantitative data collection, this total 15‐month intervention period was evidently not long enough. Action items continued to be implemented during the maintenance phase of the trial and thereafter, indicating ongoing momentum and potential for sustainability, which is supported by the finding of improved secondary prophylaxis delivery in the maintenance period compared with the intervention period. Other CQI interventions in the NT have found the number of years of participation in CQI audit cycles to be a main determinant of success.^19^

Regarding difficulties in implementing action items, particularly those to effectively engage clients (self‐management support) and the community (community linkages), barriers included lack of time available for healthcare providers to undertake these time‐consuming tasks and sociocultural/linguistic disconnection between largely non‐Aboriginal healthcare systems and Aboriginal people. Clinicians are generally trained to operate within the confines of a clinic according to their medical and nursing training; it is perhaps unreasonable to expect clinicians (often short‐term and acute‐care focused) to operate outside their usual scope of clinical practice to take a lead role in forging community linkages, especially amid competing demands on time. Yet Aboriginal clients consistently articulate that they feel alienated by the very services intended to care for them. Quotations from the client and healthcare provider interviews related earlier provide clear examples of failures in implementation of genuine self‐management support. Major revisions in models of care are needed in the Australian NT context to better meet the needs of Aboriginal people.^20^, ^21^ Healthcare providers operating in this culturally and linguistically unique environment require far more training about how to engage effectively with their clients. The value of Aboriginal governance and leadership within healthcare services and research has recently been emphasized.^22^ Our trial had Aboriginal leadership among the initial investigator team, but their involvement could not be continued because of high competing priorities—a problem frequently encountered by in‐demand Aboriginal researchers. Follow‐up studies are now under way working closely with Aboriginal researchers to foster community linkages in ARF/RHD research.

Regarding implementation challenges, we aimed to support action items that would durably embed change in medical records software (recall systems, flagging ARF/RHD patients and disease severity, supporting linkage of electronic health records) and sought to engage clinic management in CQI feedback. However, staff knowledge and education (including completing training in ARF/RHD management) is nondurable when staff are replaced; this presents an ongoing challenge and is a well‐recognized problem. A recent study found that only 20% of nurses and Aboriginal healthcare providers remained working at surveyed remote clinics 12 months after commencing, with half having left within 4 months.^23^ Understaffing and high turnover also affects the “readiness for change” of health systems, and the acceptability of quality‐improvement projects such as this one, which require active, long‐term participation from healthcare providers. In part because of staff turnover, not all sites clearly understood the trial's methods and the role of the project officers despite a thorough baseline consultation and consent process and efforts by the project team to communicate effectively with participating sites though trial newsletters, printed trial materials, face‐to‐face workshops, telephone support, and email.

Limitations of this evaluation include that serial interviews with the same healthcare provider to track evolving attitudes to the project were rarely possible given staff turnover; only 24 clinic staff members of the original 107 interviewed were available for a follow‐up interview. The nested ethnographic study that provided the patient data was essentially a convenience sample based on accessibility of the community or language spoken; this approach may have limited the diversity of data captured. The 4 sites selected differed greatly from each other in their historical, cultural, and language characteristics, but the data obtained should not be considered to represent patient views across all 10 study sites. The ethnographic component was chiefly able to provide data on experiences of ARF/RHD and health care in general, rather than impact of the project per se.

Findings of this evaluation have high relevance for delivery of ARF and/or RHD care internationally. First, that CQI interventions need to be implemented for an adequate duration to effect change in primary care settings is shown here, supported by other recent studies applying CQI processes.^19^, ^24^ Second, this evaluation illustrates the critical importance of cultural competence within health systems, especially when tackling diseases of disparity such as RHD, which predominantly affects Indigenous and disadvantaged populations globally. Third, use of the CCM to guide the delivery of care for RHD and other chronic conditions has international relevance; this intervention provides a range of examples of activities to consider across the CCM streams. We applied the CCM to delivery of secondary prophylaxis, but elements of this model also apply to primary prevention: Successful programs implemented to reduce RHD rates in the United States in the 1970s,^25^ in Cuba between the 1980s and 2000s,^26^ and in the French Caribbean islands in the 1990s^27^ were achieved using comprehensive strategies that included examples of community linkages such as awareness‐raising campaigns for affected populations. More recently, school‐based primary prevention programs (also an example of community linkages) using active case finding and school‐based delivery of treatment for streptococcal infections have been reported to be associated with reduced ARF rates.^28^ Comprehensive approaches at all public health levels from primordial through primary, secondary, and tertiary are highly applicable to RHD prevention. We are now testing a model of care in which Aboriginal community workers are employed as “care navigators” to work at the interface between medical services and families affected by RHD, to support adherence and other aspects of care.^29^

## Conclusion

By applying a comprehensive mixed‐methods evaluation to this trial, we were able to discern that the program theory underpinning the study appeared sound, but the limited impact made by the study on adherence was attributable to complex implementation challenges. Secondary prevention of ARF recurrences with penicillin prophylaxis is vital to reduce the global burden of RHD, but delivery of this regimen poses substantial challenges for health systems and clients. The multifaceted intervention we implemented was associated with some important gains, including improved adherence in a subset of clients and sustainability of health system‐strengthening approaches at some participating sites, but it did not improve adherence overall. Improving chronic condition management in primary care requires a comprehensive approach to chronic care management, especially to activities fostering client and community engagement. These evaluation findings provide strong support for new models of patient‐centered care to be implemented in Australian Aboriginal communities and in all settings with high burdens of ARF and RHD.

## Sources of Funding

This study was funded by the Australian National Health and Medical Research Council (NHMRC) project grant 1027040 and Center of Research Excellence 1080401 and by the Wesfarmers Center for Vaccines and Infectious Diseases at Telethon Kids Institute. Ralph and Maguire are supported by NHMRC fellowships (1142011 and 1046563, respectively).

## Disclosures

None.

## References

[1] Watkins DA , Johnson CO , Colquhoun SM , Karthikeyan G , Beaton A , Bukhman G , Forouzanfar MH , Longenecker CT , Mayosi BM , Mensah GA , Nascimento BR , Ribeiro ALP , Sable CA , Steer AC , Naghavi M , Mokdad AH , Murray CJL , Vos T , Carapetis JR , Roth GA . Global, regional, and national burden of rheumatic heart disease, 1990–2015. N Engl J Med. 2017;377:713–722. 28834488 10.1056/NEJMoa1603693

[2] Ralph AP , Fittock M , Schultz R , Thompson D , Dowden M , Clemens T , Parnaby MG , Clark M , McDonald MI , Edwards KN , Carapetis JR , Bailie RS . Improvement in rheumatic fever and rheumatic heart disease management and prevention using a health centre‐based continuous quality improvement approach. BMC Health Serv Res. 2013;13:525. 24350582 10.1186/1472-6963-13-525 PMC3878366

[3] Ralph AP , Read C , Johnston V , de Dassel JL , Bycroft K , Mitchell A , Bailie RS , Maguire GP , Edwards K , Currie BJ , Kirby A , Carapetis JR . Improving delivery of secondary prophylaxis for rheumatic heart disease in remote Indigenous communities: study protocol for a stepped‐wedge randomised trial. Trials. 2016;17:51. 26818484 10.1186/s13063-016-1166-y PMC4729116

[4] Ralph AP , de Dassel JL , Kirby A , Read C , Mitchell AG , Maguire GP , Currie BJ , Bailie RS , Johnston V , Carapetis JR . Improving delivery of secondary prophylaxis for rheumatic heart disease in a high‐burden setting: outcome of a stepped‐wedge, community, randomized trial. J Am Heart Assoc. 2018;7:e009308 DOI: 10.1161/JAHA.118.009308. 30018165 PMC6064833

[5] Wagner EH , Austin BT , Davis C , Hindmarsh M , Schaefer J , Bonomi A . Improving chronic illness care: translating evidence into action. Health Aff (Millwood). 2001;20:64–78. 11816692 10.1377/hlthaff.20.6.64

[6] RHDAustralia (ARF/RHD writing group) . The Australian guideline for prevention, diagnosis and management of acute rheumatic fever and rheumatic heart disease (2nd edition). National Heart Foundation of Australia and the Cardiac Society of Australia and New Zealand 2012 Available at: http://www.rhdaustralia.org.au/sites/default/files/guideline_0.pdf. Accessed July 1, 2018.

[7] Katzenellenbogen JM , Ralph AP , Wyber R , Carapetis JR . Rheumatic heart disease: infectious disease origin, chronic care approach. BMC Health Serv Res. 2017;17:793. 29187184 10.1186/s12913-017-2747-5 PMC5708129

[8] Sanson‐Fisher RW , Bonevski B , Green LW , D'Este C . Limitations of the randomized controlled trial in evaluating population‐based health interventions. Am J Prev Med. 2007;33:155–161. 17673104 10.1016/j.amepre.2007.04.007

[9] Copas AJ , Lewis JJ , Thompson JA , Davey C , Baio G , Hargreaves JR . Designing a stepped wedge trial: three main designs, carry‐over effects and randomisation approaches. Trials. 2015;16:352. 26279154 10.1186/s13063-015-0842-7 PMC4538756

[10] McCalman J , Bailie R , Bainbridge R , McPhail‐Bell K , Percival N , Askew D , Fagan R , Tsey K . Continuous quality improvement and comprehensive primary health care: a systems framework to improve service quality and health outcomes. Front Public Health. 2018;6:76. 29623271 10.3389/fpubh.2018.00076 PMC5874897

[11] RHD Australia . E‐learning: Health worker modules, Clinician modules, Administering bicillin. 2015 Available at: https://www.rhdaustralia.org.au/arf-rhd-guideline. Accessed July 1, 2018.

[12] RHD Australia . iPhone and Android Apps. 2015 Available at: http://www.rhdaustralia.org.au/apps. Accessed July 1, 2018.

[13] Chen H‐T . Program theory In: Mathison S, ed. Encyclopedia of Evaluation. Thousand Oaks: Sage Publications; 2004: 340–342.

[14] Guest G , MacQueen K , Namey EI . Introduction to applied thematic analysis In: Guest G, MacQueen K, Namey EI, eds. Applied Thematic Analysis. Thousand Oaks: Sage Publications Inc.; 2012:3–20.

[15] Sharpe G . A review of program theory and theory‐based evaluations. Am Int J Contemp Res. 2011;3:72–75.

[16] Gale NK , Heath G , Cameron E , Rashid S , Redwood S . Using the framework method for the analysis of qualitative data in multi‐disciplinary health research. BMC Med Res Methodol. 2013;13:117. 24047204 10.1186/1471-2288-13-117 PMC3848812

[17] Coleman K , Austin BT , Brach C , Wagner EH . Evidence on the Chronic Care Model in the new millennium. Health Aff (Millwood). 2009;28:75–85. 19124857 10.1377/hlthaff.28.1.75 PMC5091929

[18] Pearson ML , Wu S , Schaefer J , Bonomi AE , Shortell SM , Mendel PJ , Marsteller JA , Louis TA , Rosen M , Keeler EB . Assessing the implementation of the chronic care model in quality improvement collaboratives. Health Serv Res. 2005;40:978–996. 16033488 10.1111/j.1475-6773.2005.00397.x PMC1361183

[19] Matthews V , Schierhout G , McBroom J , Connors C , Kennedy C , Kwedza R , Larkins S , Moore E , Thompson S , Scrimgeour D , Bailie R . Duration of participation in continuous quality improvement: a key factor explaining improved delivery of Type 2 diabetes services. BMC Health Serv Res. 2014;14:578. 25408165 10.1186/s12913-014-0578-1 PMC4243284

[20] Wakerman J , Humphreys JS , Wells R , Kuipers P , Entwistle P , Jones J . Primary health care delivery models in rural and remote Australia: a systematic review. BMC Health Serv Res. 2008;8:276. 19114003 10.1186/1472-6963-8-276 PMC2642801

[21] Weeramanthri T , Hendy S , Connors C , Ashbridge D , Rae C , Dunn M , Fittock M , Cleary J , O'Donohoe L , Morton S , Swanson N . The Northern Territory preventable chronic disease strategy—promoting an integrated and life course approach to chronic disease in Australia. Aust Health Rev. 2003;26:31–42. 15368818 10.1071/ah030031

[22] Australian Institute of Aboriginal and Torres Strait Islander Studies & The Lowitja Institute . Changing the Narrative in Aboriginal and Torres Strait Islander Health Research: Four Cooperative Research Centres and the Lowitja Institute: The story so far. 2017 Available at: https://www.lowitja.org.au/sites/default/files/docs/Changing-the-narrative.pdf. Accessed July 1, 2018.

[23] Russell DJ , Zhao Y , Guthridge S , Ramjan M , Jones MP , Humphreys JS , Wakerman J . Patterns of resident health workforce turnover and retention in remote communities of the Northern Territory of Australia, 2013–2015. Hum Resour Health. 2017;15:52. 28810919 10.1186/s12960-017-0229-9 PMC5558760

[24] Schierhout G , Hains J , Si D , Kennedy C , Cox R , Kwedza R , O'Donoghue L , Fittock M , Brands J , Lonergan K , Dowden M , Bailie R . Evaluating the effectiveness of a multifaceted, multilevel continuous quality improvement program in primary health care: developing a realist theory of change. Implement Sci. 2013;8:119. 24098940 10.1186/1748-5908-8-119 PMC4124892

[25] Gordis L . Effectiveness of comprehensive‐care programs in preventing rheumatic fever. N Engl J Med. 1973;289:331–335. 4740466 10.1056/NEJM197308162890701

[26] Nordet P , Lopez R , Duenas A , Sarmiento L . Prevention and control of rheumatic fever and rheumatic heart disease: the Cuban experience (1986–1996–2002). Cardiovasc J Afr. 2008;19:135–140. 18568172 PMC3974561

[27] Bach JF , Chalons S , Forier E , Elana G , Jouanelle J , Kayemba S , Delbois D , Mosser A , Saint‐Aime C , Berchel C . 10‐year educational programme aimed at rheumatic fever in two French Caribbean islands. Lancet. 1996;347:644–648. 8596378 10.1016/s0140-6736(96)91202-7

[28] Lennon D , Anderson P , Kerdemelidis M , Farrell E , Mahi SC , Percival T , Jansen D , Stewart J . First presentation acute rheumatic fever is preventable in a community setting: a school based intervention. Pediatr Infect Dis J. 2017;36:1113–1118. 28230706 10.1097/INF.0000000000001581

[29] Menzies School of Health Research . SP Plus: expanding RHD prevention. 2017 Available at http://www.menzies.edu.au/SPPlus. Accessed July 1, 2018.

